# Standardized Tip-Apex Distance (STAD): a modified individualized measurement of cephalic fixator position based on its own femoral head diameter in geriatric intertrochanteric fractures with internal fixation

**DOI:** 10.1186/s12891-023-06286-0

**Published:** 2023-03-13

**Authors:** Yun-fa Yang, Jian-wen Huang, Xiao-sheng Gao, Zhong-he Xu

**Affiliations:** Department of Orthopaedic Surgery, the Second Affiliated Hospital, School of Medicine, Guangzhou First People’s Hospital, South China University of Technology, 1 Panfu Road, 510180 Guangzhou, Guangdong China

**Keywords:** Standardized tip-apex Distance, Intertrochanteric fracture, Internal fixation, Cut-out, The elderly

## Abstract

**Objective:**

To design a standardized Tip-Apex Distance (STAD) and analyze the clinical significance of STAD in predicting cut-out in geriatric intertrochanteric fractures with internal fixation.

**Methods:**

Firstly, we designed STAD according to the rule of TAD. We measured the STAD individually based on its own femoral head diameter (*i*FHD) instead of the known diameter of the lag screw in calculating TAD, resulting in that the STAD is simply the relative quantitation relationship of *i*FHD (the times of *i*FHD). In this study, we assumed that all the *i*FHD was 6*D* (1*i*FHD = 6*D, or* 1*D* = 1/6 of *i*FHD) in order for complete match of the Cleveland zone system, easy comparison of the STAD, and convenient identification for artificial intelligence. Secondly, we calculated and recorded all the STAD of cephalic fixator in 123 eligible ITF patients. Thirdly, we grouped all the ITF patients into the Failure and Non-failure groups according to whether cut-out or not, and analyzed the correlation between the cut-out and the STAD.

**Results:**

Cleveland zone, Parker’s ratio (AP), TAD, and STAD were associated with the cut-out in univariate analysis. However, only STAD was the independent predictor of the cut-out by multivariate analysis. No cut-out was observed when STAD ≤ 2*D* (1/3 of *i*FHD). The Receiver Operating Characteristic (ROC) curve indicated that STAD was a reliable predictor of cut-out, and the best cut-off value of STAD was 2.92*D*. Cut-out rate increased dramatically when STAD increased, especially when STAD > 3*D* (1/2 of *i*FHD).

**Conclusion:**

Essentially, the STAD is a relative quantitation relationship of *i*FHD. The STAD is a reliable measurement of cephalic fixator position in predicting cut-out in geriatric ITF patients with single-screw cephalomedullary nail fixations. For avoiding cut-out, the STAD should be no more than a half of *i*FHD.

**Level of evidence:**

Level III, Prognostic Study

## Introduction

With the aging population, femoral intertrochanteric fracture (ITF) is increasingly common [1]. ITF lead to great burdens to the whole society because ITF is associated with bed-ridden related complications, cognitive difficulties, and high mortality [1–3]. Nowadays, ITF is treated surgically with internal fixation, particularly with the cephalomedullary nail (CMN) [4]. However, implant failures, including cut-out, cut-through, and implant breakage, remain a challenge to orthopedists despite the progress of surgical procedures and implant modifications [5–7]. Especially some implant failures such as cut-out need reoperation, have great harms to these aging patients.

It is a consensus that cut-out is associated with the position of cephalic fixator, and cut-out is commonly measured by the tip-apex distance (TAD, an absolute measurement value of cephalic fixator position based on *D*_*true*_ (the known diameter of the lag screw)), which is still a classical predictor of the cut-out of cephalic fixator [8–14]. However, it is still unknown how much the exact TAD should be so as to prevent cut-out. Baumgaertner found that lower risk of the cut-out in the cases of “TAD < 25 mm” [8]. But subsequently, some studies argued that TAD could be much bigger (< 30 mm) or should be much smaller (< 20 mm, or even < 15 mm) for avoiding cut-out [10–12]. Thereafter, in order to remedy the weakness of TAD, many scholars have sought several new methods, such as calcar-referenced tip-apex-distance (CalTAD) and tip-neck distance ratio (TNDR), for evaluating cephalic fixator placement [15–17]. Both CalTAD and TNDR favor relatively inferior placement on the AP view and posterior placement on the lateral view. However, some clinical studies have confirmed that CalTAD is not superior to TAD in predicting cut-out [12, 14, 18]. TNDR has also not enough consideration on the depth of the cephalic fixator, while we know that the depth is a very important parameter for optimal nail position. Consequently, TAD, which favors a central and subcortical cephalic fixator placement within the femoral head, is still a generally recognized criterion for cephalic fixator placement in clinical practice. So far, it is still unknown how much the exact TAD should be in order for preventing cut-out.

We speculate that the reason might be the great diversity of femoral head diameter (FHD) in different body height, genders and races [19–23]. Mokrovic revealed that there were significant differences in femoral geometry between various ethnic groups [21]. They found that the differences of FHD could be up to 22 mm (varying from 30 to 52 mm) [21]. Besides gender and ethnics, the FHD also varied a lot from diverse regions even in the same country (the FHD in Southeast China and North China population were 45.40 ± 3.21 mm and 51.03 ± 3.88 mm, respectively) [20, 23]. Furthermore, FHD has a good correlation with body height [19–23]. Some finite element analysis also confirmed that TAD should be individually adjusted [24–26].

Therefore, we hypothesize that the standardized tip-apex distance (STAD, a relative measurement of TAD), which was designed according to the rule of TAD and individually measured based on its own FHD (*i*FHD), was more appropriate for predicting cut-out than the absolute measurement of TAD did. Therefore, we aim to (1) design STAD for individualized measurement of cephalic fixator position based on *i*FHD instead of the known diameter of the lag screw (*D*_*true*_) for geriatric ITF with internal fixation; and (2) verify the correlation between the STAD and the cut-out.

## Methods

### STAD and its measurement

In this study, STAD was calculated according to the rule of TAD and STAD was individually measured based on its own FHD (*i*FHD) instead of the known diameter of the lag screw (*D*_*true*_) in calculating TAD [8], resulting in that the STAD is simply the quantitation relationship of *i*FHD (the times of *i*FHD).

We defined the femoral head as a regular sphere with the *i*FHD of 6*D* (1*i*FHD = 6*D, or* 1*D* = 1/6 of *i*FHD) in order for good match of Cleveland zone and convenient comparison of STAD. That was to say we assumed all the *i*FHD as “6*D*” no matter how big the *i*FHD truely was. Because in Cleveland zone system, the femoral head was defined as a regular sphere and divided into superior, central, and inferior thirds on the anteroposterior radiograph and into anterior, central, and posterior thirds on the lateral radiograph, resulting in the femoral head is divided into nine separate zones for evaluating the cephalic fixator tip position [27]. When we defined “1*i*FHD = 6*D*”, the radius of the femur would be 3*D*, and 1/12, 2/12, 3/12, 4/12, 5/12, 6/12, 7/12, … and 12/12 times of *i*FHD would be 0.5*D*, 1*D*, 1.5*D*, 2*D*, 2.5*D*, 3*D*, 3.5*D*, …, and 6*D*, respectively.

**Fig. 1:**
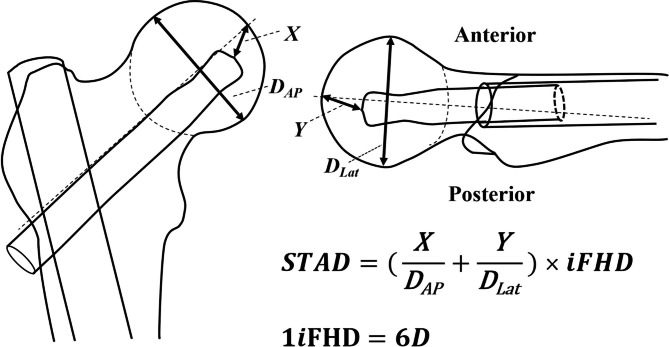
An illustration about the measurement of STAD. STAD was calculated according to the rule of TAD, while STAD was measured based on the its own FHD (*i*FHD) instead of *D*_*true*_ in caculating TAD. The femoral head was considered as a regular sphere. All the *i*FHD was assumed the same constant value (in this study, 1*i*FHD = 6*D*, or 1*D* = 1/6 *i*FHD, no matter how big the actual *i*FHD was) in order for easier calculation and more convenient comparison. According to the rule of TAD, STAD was the sum of the actually measured distance of the nail tip to the apex of femoral head (*X*) divided the actually measured FHD (*D*_*AP*_) on the anteroposterior view (*X/D*_*AP*_) and the actually measured distance of the nail tip to the apex of femoral head (*Y*) divided the actually measured FHD (*D*_*Lat*_) on the lateral view (*Y*/*D*_*Lat*_), respectively, then multiplied *i*FHD. Since STAD was calculated according to the principle of TAD and measured based on the its own FHD (*i*FHD). When we define “1*i*FHD = 6*D*”, the radius of the femur head will be 3*D*, and 1/12, 2/12, 3/12, 4/12, 5/12, 6/12, 7/12, … and 12/12 times of *i*FHD will be 0.5*D*, 1*D*, 1.5*D*, 2*D*, 2.5*D*, 3*D*, 3.5*D*, …, and 6*D*, respectively. So, STAD = 3*D* meant that the STAD was a half of its own FHD

Since STAD was calculated according to the principle of TAD and STAD was individually measured based on *i*FHD instead of the known diameter of the lag screw. Therefore, STAD can be individually compared. According to the principle of TAD, we defined that the STAD was the sum of the actually measured distance of the cephalic fixator tip to the apex of femoral head (*X*) divided the actually measured FHD (*D*_*AP*_) on anteroposterior view (*X/D*_*AP*_) and the actually measured distance of the cephalic fixator tip to the apex of femoral head (*Y*) divided the actually measured FHD (*D*_*Lat*_) on lateral view (*Y*/*D*_*Lat*_) then multiplied *i*FHD. Since *X*, *D*_*AP*_ and *Y*, *D*_*Lat*_ were actually measured in the same radiography on AP view and lateral view respectively, therefore, the value of *X/D*_*AP*_ and *Y/D*_*Lat*_ in any one patient would be constant individually, no matter how much the magnification was. Since STAD was calculated according to the principle of TAD and measured based on *i*FHD (STAD was several times of *i*FHD) and when *i*FHD = 6*D*, STAD = 3*D* meant that the STAD was a half of *i*FHD (Fig. 1).

## Primary verification of STAD in geriatric ITF patients

**Fig. 2:**
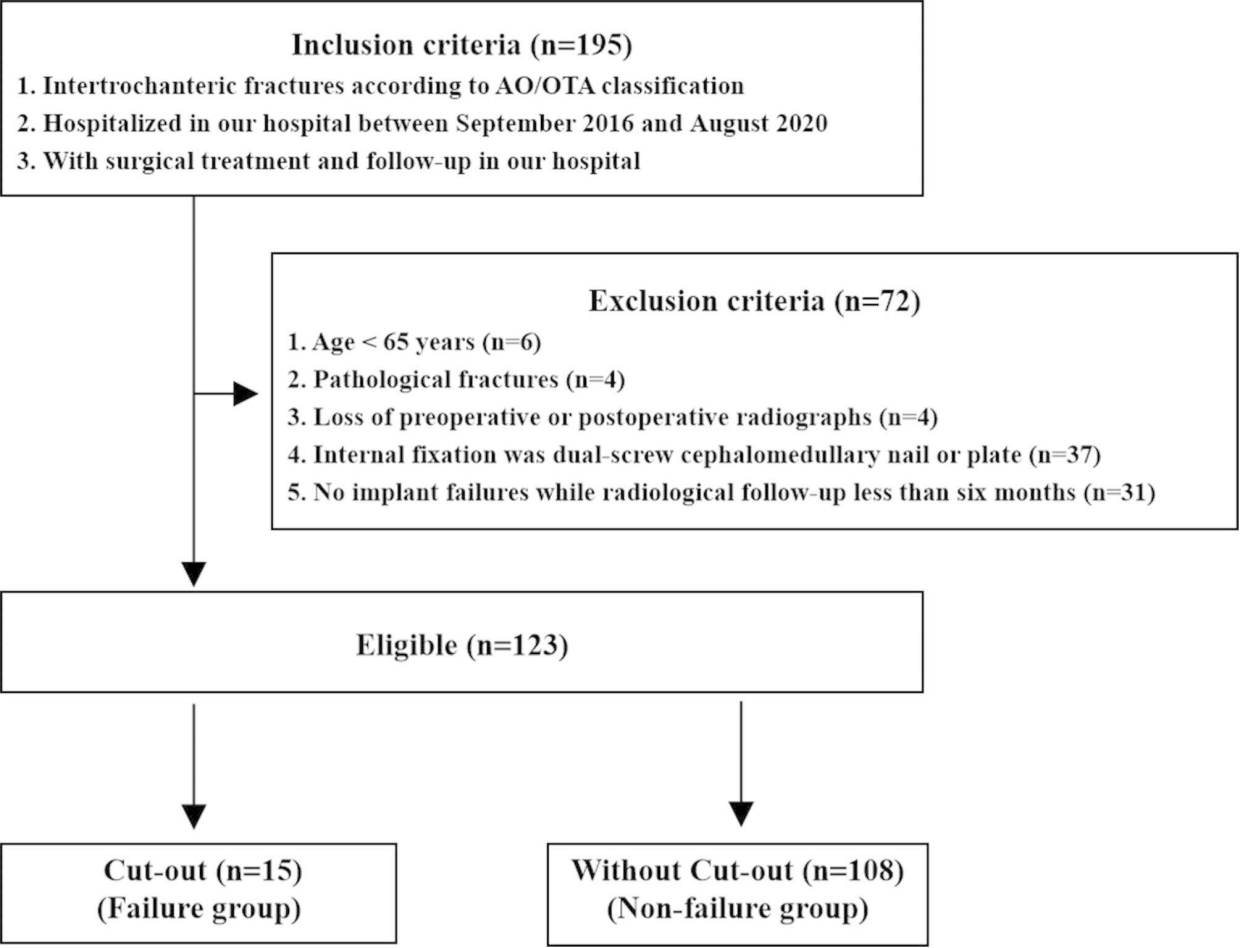
Flow of patients through the study

In order for primary verification of STAD in geriatric ITF patients, we collected the geriatric ITF patients as many as possible. There were 195 ITF patients with surgical treatment and follow-up in our hospital between September 2016 and August 2020 (Fig. 2). Exclusion criteria: (1) age < 65 years, (2) pathological fractures, (3) loss of preoperative or postoperative radiographs, (4) internal fixation was dual-screw cephalomedullary nail or plate fixation, (5) patients with no implant failures while radiological follow-up less than six months.

Totally, there were 123 eligible patients including 43 males and 80 females with a mean age of 80.4 ± 8.4 years in this study. The mean follow-up was 11.2 months (range, 6–24 months). All the eligible ITF patients were divided into the Failure group and Non-failure group according to whether cut-out or not. Cut-out was regarded as the upper extrusion of the cephalic fixator from the femoral head. Overall, 15 ITF patients were observed with cut-out. Clinical data including age, gender, fracture site, fracture classifications, American Society of Anesthesiologists (ASA) classification, bone quality, anesthesia, fixation type, reduction quality, Cleveland zone [27], Parker’s ratio [28], TAD, and STAD were collected and analyzed.

Fracture classification was determined by preoperative radiographs according to the AO/OTA system (2018 version) [29]. Bone quality was evaluated by the Singh index in preoperative AP radiographs [30]. Reduction quality was graded into three groups including good, acceptable, and poor reduction based on the criterion developed by Baumgaertner [31]. Central-central or inferior-central nailing position according to the Cleveland Zone system (Zone 5 or Zone 8) was regarded as acceptable placement. Parker’s ratio and TAD were as measured as described in the literature [8, 28]. All of the radiological parameters were blindly evaluated by two observers (JWH & XSG).

### Statistical analysis

The occurrence of cut-out was defined as the dependent variable. Univariate analysis of continuous and categorical variables was performed with Student’s *t*-test and Chi-square test, respectively. All of the significant variables in univariate analysis (*p* < 0.1) and potential variables were selected into a multivariate logistic model. TAD and STAD were considered as both continuous variables and categorical variables being selected into two respective models. The intraclass correlation coefficient (ICC) for continuous variables by a two-way random-effects model and κ coefficient for categorical variables were used for the consistency test. All analyses above were performed using SPSS (IBM SPSS Statistic for Windows, Version 25.0. Armonk, NY: IBM Corp). All tests were two-sided, and the statistical significance was defined as the p-value below 0.05. The receiver operating characteristic (ROC) curves were performed to assess cut-off value and the reliability of STAD in predicting cut-out with MedCalc® Statistical Software version 19.5.6 (MedCalc Software Ltd, Ostend, Belgium; https://www.medcalc.org; 2020).

## Results

**Fig. 3:**
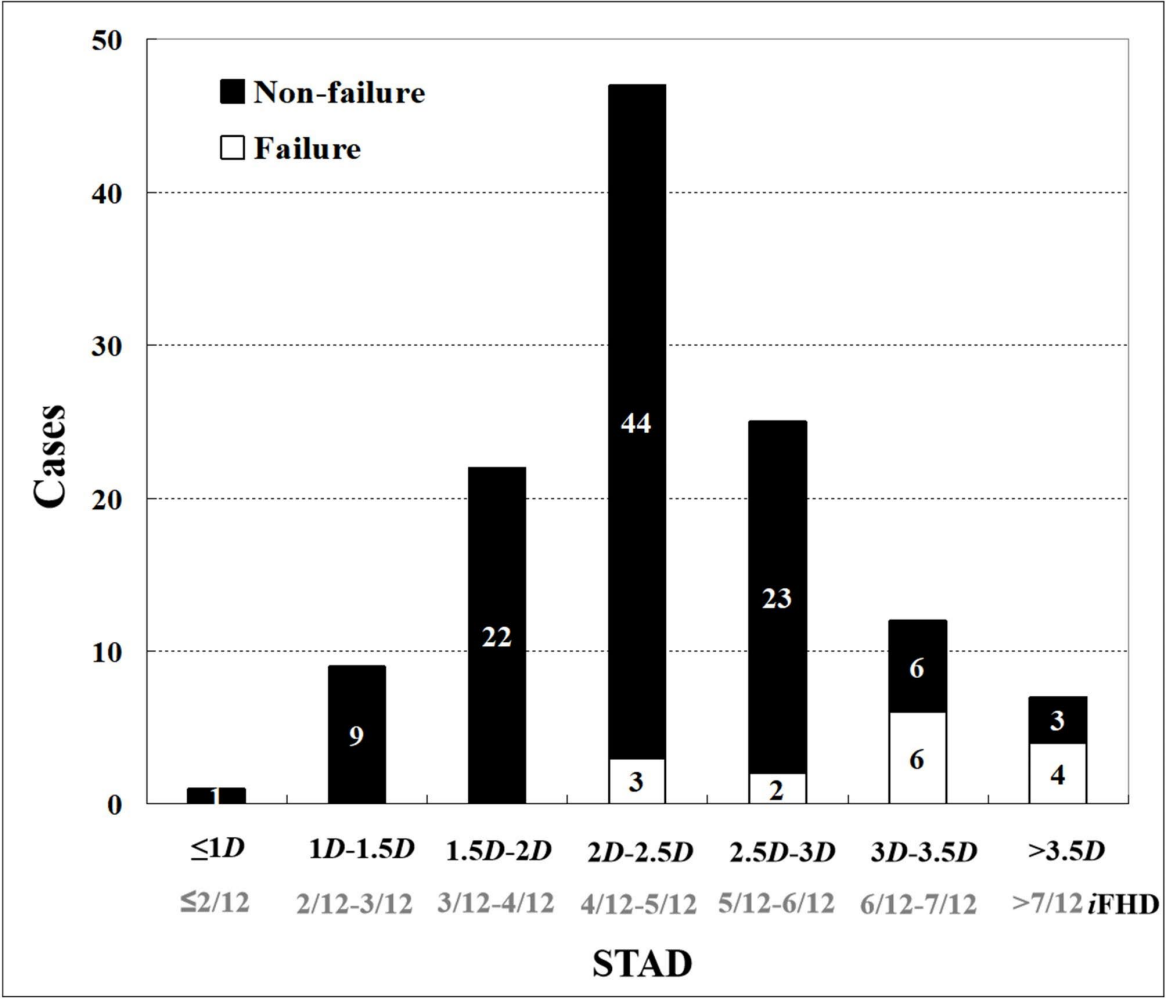
The distributions of STAD in different intervals. The histogram shown that no cut-out were observed with “STAD ≤ 2*D* (4/12 times of *i*FHD)” (0/32), and cut-out rate is higher with the increasing of STAD [Cut-out rate: 6.4% in STAD ranged in “2*D* – 2.5*D*” (3/47), 8.0% in “2.5*D* – 3*D*” (2/25), 50.0% in “3*D* – 3.5*D*” (6/12) and 57.1% in “STAD > 3.5*D* (7/12 times of *i*FHD)” (4/7)]. STAD = 3*D* meant that the STAD was a half of its own FHD because STAD was calculated according to the rule of TAD and measured based on the its own FHD (*i*FHD) and 1*i*FHD = 6*D*.

The frequency histogram shown a normal distribution of all STAD (Fig. 3**).** Most STAD of cephalic fixators ranged in the interval of “2.0*D* – 2.5*D*” (47/123). No cut-out was observed when STAD ≤ 2*D* (0/32), and cut-out rates were 6.4% when STAD ranged in “2*D* – 2.5*D*” (3/47), 8.0% in “2.5*D* – 3*D*” (2/25), 50.0% in “3*D* − 3.5*D*” (6/12), and 57.1% in STAD > 3.5*D* (4/7), respectively.

In the univariate analysis (Table 1), no significant differences were found in age, gender, fracture site, AO/OTA fracture classification, Singh index, anesthesia, ASA classification, fixation type and reduction quality between the Failure group (with cut-out) and non-failure group (without cut-out) (p > 0.05). While statistically significant differences were observed in Cleveland zone, Parker’s ratio (AP), TAD, and STAD between the Failure group (with cut-out) and non-failure group (without cut-out). Unacceptable Cleveland zone (*p* = 0.002), higher Parker’s ratio (AP) (*p* = 0.043), TAD (*p* < 0.001), and STAD (*p* < 0.001) were significantly associated with cut-out. Furthermore, after considering TAD and STAD as categorical variables, significant differences of cut-out remain in the ITF patients with “TAD > 25 mm” (*p* = 0.022), or with “STAD > 3*D*” (*p* < 0.001), respectively.

**Table 1:** Univariate analysis of clinical data

Factor	Overall (n = 123)	without Cut-out group (n = 108)	with Cut-out group (n = 15)	p-value	OR (95% CI)
Age, years	80.4 ± 8.40	80.3 ± 8.43	81.1 ± 8.46	0.744^†^	1.01 (0.95 to 1.08)
Gender (male/female), n	43/80	40/68	3/12	0.255^*^	2.35 (0.63 to 8.84)
Fracture site (left/right), n	72/51	64/44	8/7	0.781^*^	1.27 (0.43 to 3.77)
AO/OTA classification, n (%)				0.108^*^	NA
31A1	62 (50.4)	58 (53.7)	4 (26.7)		
31A2	56 (45.5)	46 (42.6)	10 (66.7)		
31A3	5 (4.1)	4 (3.7)	1 (6.6)		
Singh index, n				0.428^*^	1.56 (0.52 to 4.68)
≤ 3 / > 3	62/61	53/55	9/6		
ASA score, n				0.956^*^	1.03 (0.34 to 3.10)
≤ 2 / >2	50/73	44/64	6/9		
Anesthesia (spinal/general), n	95/28	82/26	13/2	0.517^*^	2.06 (0.44 to 9.74)
Fixation type (blade/screw), n	41/82	35/73	6/9		
Reduction quality, n (%)				0.176^*^	NA
Good	54 (43.9)	50 (46.3)	4 (26.7)		
Acceptable	47 (38.2)	38 (35.2)	9 (60.0)		
Poor	22 (17.9)	20 (18.5)	2 (13.3)		
Cleveland zone, n				**0.002** ^*^	6.39 (1.99 to 20.57)
Acceptable^&^/the others	105/18	97/11	8/7		
Parker’s ratio (AP), %	48.57 ± 8.19	48.01 ± 7.48	52.64 ± 11.67	**0.043** ^**†**^	1.07 (1.00 to 1.15)
Parker’s ratio (Lat), %	49.82 ± 8.19	49.59 ± 7.56	51.46 ± 11.99	0.410^†^	1.02 (0.96 to 1.10)
TAD, mm	19.16 ± 4.97	18.55 ± 4.73	23.57 ± 4.53	**< 0.001** ^**†**^	1.23 (1.09 to 1.40)
TAD, n					
> 25 mm / < 25 mm	20/103	14/94	6/9	**0.022** ^*^	4.48 (1.38 to 14.51)
STAD, *D*	2.35 ± 0.64	2.24 ± 0.56	3.14 ± 061	**< 0.001** ^**†**^	11.98 (3.78 to 37.94)
STAD, n	**§**	**§**	**§**	**< 0.001** ^*^	NA

^†^ Student’s t-test

^*^ Chi-square test

NA, not applicable

§ The distribution of values is indicated in Fig. 3

^&^ Acceptable means Cleveland zone 5 or 8

OR, odds ratio; CI, confidence interval; AO/OTA, AO Foundation and Orthopaedic Trauma Association; ASA, American Society of Anesthesiologists; AP, anteroposterior view; Lat, lateral view; TAD, tip-apex distance; STAD, standardized tip-apex distance; *i*FHD, its own femoral head diameter; *i*FHD = 6*D* in this study

In multivariate analysis (Table 2), unstable fractures, Cleveland zone, reduction quality, Parker’s ratio (AP), TAD (or “TAD > 25 mm”), and STAD (or “STAD > 3*D*”) were entered into binary logistic models. In the two models, unstable fractures, Cleveland zone, reduction quality, Parker’s ratio (AP), and TAD were no longer associated with cut-out, and the statistical difference was only observed in STAD. In the model considering STAD and TAD as continuous variables, a bigger STAD had significant association with cut-out (Adjusted OR = 23.312, 95% CI: 2.649–205.179, *p* = 0.005). In the model considering STAD and TAD as categorical variables, there is also a significant increasing risk of cut-out when “STAD > 3*D*” (Adjusted OR = 20.713, 95% CI: 3.846–111.551, *p* < 0.001).

**Table 2:** Multivariate logistic regression analysis

Factor	*p*-value*	Adjusted OR* (95% CI)	*p*-value**	Adjusted OR** (95% CI)
Unstable facture	0.198	2.730 (0.592 to 12.583)	0.179	2.932 (0.610 to 14.097)
Accept reduction	0.283	0.435 (0.096 to 1.985)	0.297	0.431 (0.088 to 2.101)
Parker’s ratio, AP	0.407	1.033 (0.957 to 1.114)	0.663	1.017 (0.942 to 1.098)
Acceptable zone^&^	0.377	0.499 (0.107 to 2.331)	0.191	0.367 (0.082 to 1.650)
TAD	0.459	1.093 (0.863 to 1.383)	N/A	N/A
STAD	**0.005**	**23.312 (2.649 to 205.179)**	N/A	N/A
TAD > 25 mm	N/A	N/A	0.941	1.071 (0.172 to 6.670)
STAD > 3*D*	N/A	N/A	**< 0.001**	**20.713 (3.846 to 111.551)**

N/A, not applicable;

OR, odds ratio; CI, confidence interval; TAD, tip-apex-distance; STAD, standardized tip-apex distance

^&^ Not acceptable zone means Cleveland zone 1–4, 6, 7, 9

* Model considering TAD and STAD as continuous variables

** Model considering TAD and STAD as categorical variables

*i*FHD, its own femoral head diameter; *i*FHD = 6*D* in this study

**Fig. 4:**
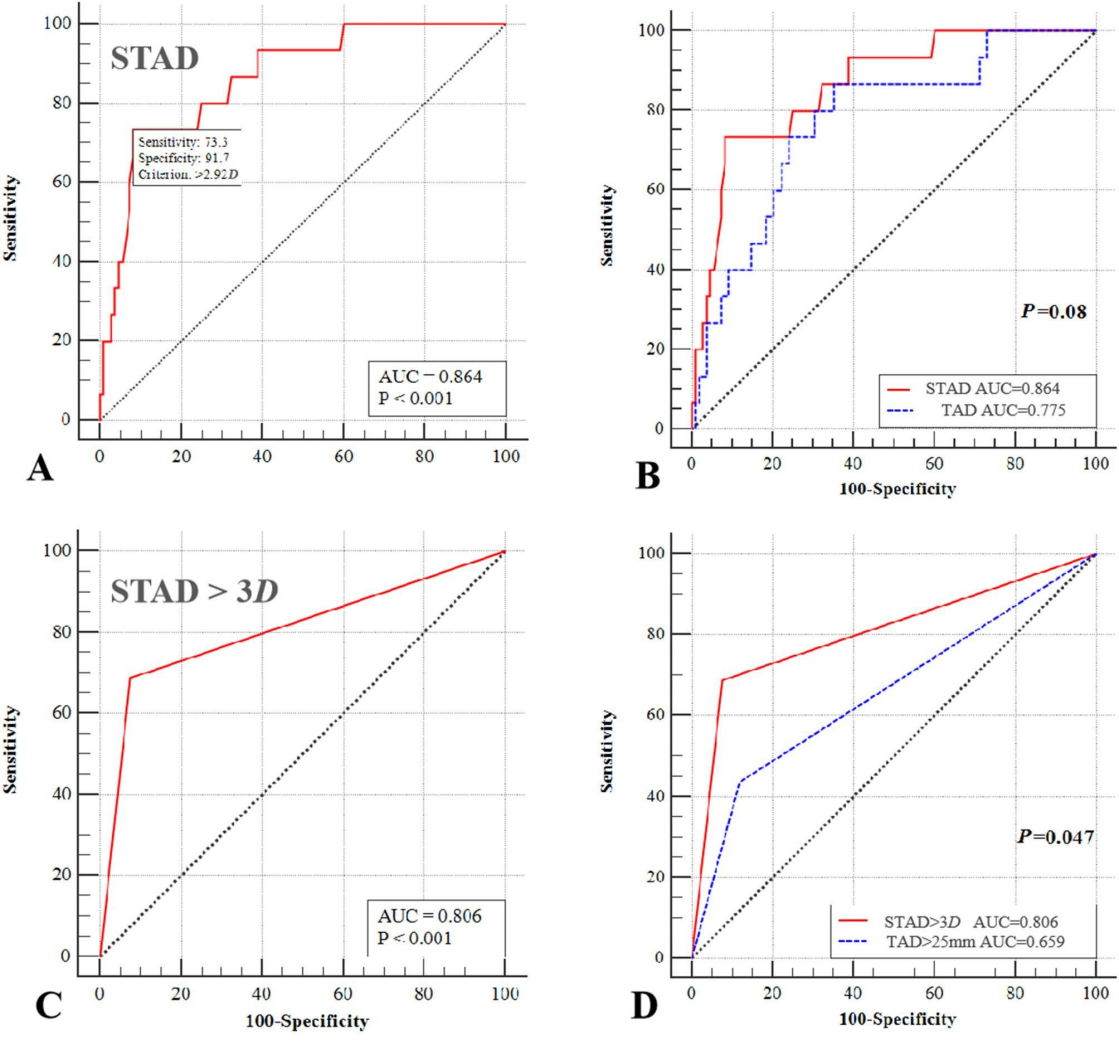
The ROC analysis indicated that STAD was a reliable measurement in predicting cut-out (Area under the curve (AUC) = 0.864, *p* < 0.001), of which the best cut-off value was 2.92*D* (Sensitivity = 73.3%, specificity = 91.7%) (A). In comparing STAD with TAD, there was no significant difference on predicting cut-out between STAD and TAD (STAD, AUC = 0.864; TAD, AUC = 0.775; *p* = 0.08) (B). However, “STAD > 3*D*” was observed with good predicted effect of cut-out (AUC = 0.806, *p* < 0.001) (C). In comparing “STAD > 3*D*” with “TAD > 25 mm”, significant difference of cut-out was found in “STAD > 3*D*” (“STAD > 3*D*”, AUC = 0.806; “TAD > 25 mm”, AUC = 0.659; *p* = 0.047) (D)

The ROC analysis (Fig. 4) indicated that STAD was a reliable measurement in predicting cut-out (Area under the curve (AUC) = 0.864, *p* < 0.001), of which the best cut-off value was 2.92*D* (sensitivity = 73.3.4%, specificity = 91.7%) (Fig. 4A). In comparing STAD with TAD, there was no significant difference on predicting cut-out between STAD and TAD (STAD, AUC = 0.864; TAD, AUC = 0.775; *p* = 0.08) (Fig. 4B). However, “STAD > 3*D*” was a good predictor of cut-out (AUC = 0.806, *p* < 0.001) (Fig. 4C). In comparing “STAD > 3*D*” and “TAD > 25 mm”, the cut-out rate was much lower in “STAD > 3*D*” (“STAD > 3*D*”, AUC = 0.806; “TAD > 25 mm”, AUC = 0.659; *p* = 0.047) (Fig. 4D).

The results of interobeserver reliability for measuring parameters were shown in Table 3. The reliabilities for Singh index, Cleveland zone and fracture classification were excellent, and those for reduction quality, Parker ratio (AP), Parker ratio (Lat), TAD and STAD were almost excellent (Table 3).

**Table 3:** Reliability between two independent observers for measuring variables

Variable	ICC or κ	95% CI	Reliability
Singh index	0.682	0.573 to 0.767	Excellent
Cleveland zone	0.669	0.565 to 0.773	Excellent
fracture classification	0.788	0.709 to 0.847	Excellent
Reduction quality	0.809	0.709 to 0.909	Almost perfect
Parker ratio (AP)	0.917	0.884 to 0.942	Almost perfect
Parker ratio (Lat)	0.924	0.893 to 0.947	Almost perfect
Tip-apex distance (TAD)	0.905	0.866 to 0.933	Almost perfect
Standardized tip-apex distance (STAD)	0.906	0.743 to 0.955	Almost perfect

AP, anteroposterior view; LAT, lateral view; CI, confidence interval; ICC, intraclass correlation coefficient; κ, Kappa coefficient

## Discussion

ITF is a great concern in the aging society due to the increasing incidence and large financial burden [1, 2]. Nowadays, CMN is the most commonly used in ITF surgeries [4], but cut-out after CMN fixation is still a great challenge to orthopedists. Appropriate position of cephalic fixator in surgery, ordinarily considered as TAD, had been proved to associate with the cut-out in numerous studies [8–14]. However, in order for effective prevention of cut-out, it is still not clear on how much the exact TAD should be. Moreover, previous studies have suggested that the optimal TAD should be adjusted individually [24–26]. Since FHD had a good correlation with body size such as height [19–23], we could reasonably standardize the *i*FHD as a constant value (1*i*FHD = 6*D*, or 1*D* = 1/6 of *i*FHD, in this study), no matter how big the *i*FHD truely is. In this study, we designed the STAD, which was calculated according to the principle of TAD and individually measured based on *i*FHD. We confirmed that STAD was a reliable measurement of cephalic fixator position in predicting cut-out in geriatric ITF patients with CMN fixations. To avoid cut-out, the STAD of the cephalic fixator should be no more than 3*D* (1/2 of *i*FHD).

### STAD is reliable for predicting cut-out

The most important finding of our study is the reliability of the STAD in predicting cut-out. We found that the cut-out occurrence rose dramatically when STAD increased. For every 1*D* (1/6 of *i*FHD) increase in STAD, the risk of cut-out increased more than 23-fold (Adjusted OR = 23.312, 95% CI: 2.649–205.179). The ROC analysis indicated a threshold of STAD at 2.92*D* (a little bit less than 1/2 of *i*FHD) had the highest Youden index. But considering the convenience in clinical usage, we further recommended a cut-off value of STAD at 3*D* ((because “*i*FHD = 6*D*”, a radius of its own femoral head was just 3*D* (1/2 of *i*FHD), very intuitive and convenient)) in predicting cut-out. The logistic regression model confirmed that there was a roughly 20 times higher risk of cut-out when “STAD > 3*D*” (Adjusted OR = 20.713, 95% CI: 3.846–111.551). In ROC analysis of correlation between “STAD > 3*D*” and cut-out, “STAD > 3*D*” was a good predictor (AUC = 0.806, *p* < 0.001). Therefore, we demonstrate that STAD should be no more than 3*D* (the tip-apex distance should be no more than a radius of its own femoral head). We also found that “STAD > 3*D*” had significant higher reliability in predicting cut-out than “TAD > 25 mm” did by ROC analysis (*p* = 0.047).

Although STAD had higher reliability for predicting cut-out than TAD, no significant differences between STAD and TAD were found (*p* = 0.08). This discrepancy may be explained by the population and the nonlinearity of variables. Firstly, the population in the current study were mostly community residents with hardly regional and racial differences, which might mean that there is scarce difference in FHD. Secondly, STAD or TAD were nonlinear variables. The increasing risk of the cut-out was different when STAD or TAD was in different ranges. Thus, more studies are necessary to make further investigation and verification.

We did not regard STAD = 2*D* (1/3 of *i*FHD) as an ideal threshold though no cut-out when “STAD < 2*D*” in this study. Firstly, the cut-off value of STAD at 2*D* was not convenient because “1*i*FHD = 6*D, or* 1*D* = 1/6 of *i*FHD” in this study. Secondly, it is clear that “2*D*” as the threshold of STAD may have low specificity in predicting cut-out, while “STAD < 2*D*” had a sensitivity of 100% and a specificity of 30.5% according to ROC analysis. Thirdly, coincided with previous studies concluding that cephalic fixator placement too close to the subchondral bone may lead to penetration through the head (cut-though) [32–34], we consciously placed the cephalic fixator deeply enough rather than too deep for avoiding cut-through.

### STAD is essentially a certain proportion of its own FHD

Since STAD is designed according to the principle of TAD and individually measured based on *i*FHD, as a result, STAD is essentially a certain proportion of *i*FHD, which has no difference caused by the height, weight, age, gender, and race. Therefore, STAD may be suitable for all kinds of people. Moreover, because STAD is individually measured based on *i*FHD (measured on the same AP or Lateral view radiograph, respectively), no matter how much the magnification is, the surgeons or artificial intelligence can even utilize STAD to estimate and adjust cephalic fixator position through fluoroscopy on AP and lateral views intraoperatively instead of the calculation and transformation of the absolute value of TAD after surgeries.

### Limitations or weaknesses

However, there are still some limitations in this study. Firstly, the measurement of STAD is based on an ideal condition that the femoral head was regarded as a regular sphere. Actually, the geometric center may be a little bit affected by body position and X-ray direction. Secondly, we only verify the applicability of STAD value in the single-screw cephalomedullary nails. As a result, the conclusion maybe not suit for other types of internal fixation. Thirdly, the ITF patients treated in our department are all Chinese with little diversity of race and region. Therefore, STAD may have no clinical promotion significance. In Addition, instead of using BMD, we just use the Singh index for assessing osteoporosis, which may result in subjective outcomes. However, osteoporosis is one of the most important causes of implant failures in elderly patients. And finally, the drawbacks of retrospective design and the limited sample size are the obvious weakness of this study. Thus, prospective clinical studies with large sample sizes are necessary to verify the clinical significance and applicability.

## Conclusion

STAD is essentially a relative quantitation relationship of *i*FHD. STAD is a reliable measurement of cephalic fixator position in predicting cut-out in geriatric ITF patients with single-screw CMN fixations. To avoid cut-out, we should at least place the cephalic fixator with “STAD ≤ 3*D*”, that is, the STAD should be no more than a half of *i*FHD (both the actual tip-apex distance of “*X*” on the AP view and “*Y*” on the lateral view should better be no more than half times of radius of its own femoral head, intraoperatively).

## Data Availability

The dataset supporting the conclusions of this study is available upon request by contacting the corresponding author, but the primary data were not shared because other studies related these primary data were underway confidentially.
